# Non-lobar atelectasis generates inflammation and structural alveolar injury in the surrounding healthy tissue during mechanical ventilation

**DOI:** 10.1186/s13054-014-0505-1

**Published:** 2014-09-09

**Authors:** Jaime Retamal, Bruno Curty Bergamini, Alysson R Carvalho, Fernando A Bozza, Gisella Borzone, João Batista Borges, Anders Larsson, Göran Hedenstierna, Guillermo Bugedo, Alejandro Bruhn

**Affiliations:** Departamento de Medicina Intensiva, Facultad de Medicina, Pontificia Universidad Católica de Chile, Santiago, Chile; Hedenstierna Laboratory, Department of Surgical Sciences, Section of Anaesthesiology & Critical Care, Uppsala University, Hospital, 751 85, Uppsala, Sweden; Laboratory of Respiratory Physiology, Federal University of Rio de Janeiro, Carlos Chagas Filho, Institute of Biophysics-CCS, Rio de Janeiro, Brazil; Laboratório de Investigação em Medicina Intensiva, IPEC, Fiocruz, RJ Brazil; Departamento de Enfermedades Respiratorias, Facultad de Medicina, Pontificia Universidad Católica de Chile, Santiago, Chile; Laboratório de Pneumologia LIM-09, Disciplina de Pneumologia, Heart Institute (Incor) Hospital das Clínicas da Faculdade de Medicina da Universidade de São Paulo, São Paulo, Brazil; Hedenstierna Laboratory, Department of Medical Sciences, Clinical Physiology, Uppsala University, Uppsala, Sweden

## Abstract

**Introduction:**

When alveoli collapse the traction forces exerted on their walls by adjacent expanded units may increase and concentrate. These forces may promote its re-expansion at the expense of potentially injurious stresses at the interface between the collapsed and the expanded units. We developed an experimental model to test the hypothesis that a local non-lobar atelectasis can act as a stress concentrator, contributing to inflammation and structural alveolar injury in the surrounding healthy lung tissue during mechanical ventilation.

**Methods:**

A total of 35 rats were anesthetized, paralyzed and mechanically ventilated. Atelectasis was induced by bronchial blocking: after five minutes of stabilization and pre-oxygenation with F_I_O_2_ = 1.0, a silicon cylinder blocker was wedged in the terminal bronchial tree. Afterwards, the animals were randomized between two groups: 1) Tidal volume (V_T_) = 10 ml/kg and positive end-expiratory pressure (PEEP) = 3 cmH_2_O (V_T_10/PEEP3); and 2) V_T_ = 20 ml/kg and PEEP = 0 cmH_2_O (V_T_20/zero end-expiratory pressure (ZEEP)). The animals were then ventilated during 180 minutes. Three series of experiments were performed: histological (n = 12); tissue cytokines (n = 12); and micro-computed tomography (microCT; n = 2). An additional six, non-ventilated, healthy animals were used as controls.

**Results:**

Atelectasis was successfully induced in the basal region of the lung of 26 out of 29 animals. The microCT of two animals revealed that the volume of the atelectasis was 0.12 and 0.21 cm^3^. There were more alveolar disruption and neutrophilic infiltration in the peri-atelectasis region than the corresponding contralateral lung (control) in both groups. Edema was higher in the peri-atelectasis region than the corresponding contralateral lung (control) in the V_T_20/ZEEP than V_T_10/PEEP3 group. The volume-to-surface ratio was higher in the peri-atelectasis region than the corresponding contralateral lung (control) in both groups. We did not find statistical difference in tissue interleukin-1β and cytokine-induced neutrophil chemoattractant-1 between regions.

**Conclusions:**

The present findings suggest that a local non-lobar atelectasis acts as a stress concentrator, generating structural alveolar injury and inflammation in the surrounding lung tissue.

## Introduction

Acute respiratory distress syndrome (ARDS) is characterized by prominent heterogeneous distribution of lung densities, and the regional transalveolar pressures may present marked dispersion [1,2]. At the microscale, when an alveolus collapses the traction forces exerted on its walls by adjacent expanded units increase and concentrate. These forces may promote its re-expansion at the expense of potentially injurious stresses at the interface between the collapsed and the expanded units [1-3]. These inhomogeneities are also known as pressure multipliers or stress risers [1,2,4,5]. This conceptual framework was described by Mead and coworkers in 1970 and is essentially related to alveolar interdependence phenomena [3,6].

It is well-known that mechanical ventilation in itself can harm the lung, inducing or aggravating ARDS. Much debate remains, however, over pivotal concepts regarding the pathophysiology of ventilator-induced lung injury (VILI) [7,8], especially about the precise contribution, kinetics, and primary role of regional putative VILI mechanisms [1,2,9,10]. Hypothesized mechanisms of VILI include low-volume injury and over-distension of the lungs. Low-volume injury encompasses local concentration of stresses in the vicinity of collapsed regions and also cyclical recruitment of airways and alveoli [3,10-14]. Over-distension injury results from increased regional lung volume and excessive deformation of epithelial and endothelial cells, as well as of the extracellular matrix, leading to a pro-inflammatory response. In some studies, low-volume injury was indicated to predominate as mechanism of damage [4,5,10-14], whereas in others including laboratory [6,10,15] and clinical studies [7,8,16,17] over-distension was considered the predominant VILI mechanism.

In the heterogeneously expanding lung, the initial triggering mechanism of local injury and inflammation may occur predominantly in regions likely to undergo either concentration of stress or cyclic recruitment. However, there are scarce data on *in vivo* topographical association between regional alveolar aeration and inflammatory changes. Recently, Borges *et al*., using a two-hit injury ARDS animal model observed that inflammation was more pronounced within the normally and poorly aerated regions [18]. These findings challenge the classical paradigm of VILI, which claims that it occurs mainly in either over-distended or collapsed lung regions. Notwithstanding, theoretical and physiological models still suggest that atelectasis may be a decisive early event [19], acting as a key stress raiser in its surrounding in the very beginning of the inflammatory process accompanying VILI [6,20].

We developed a rat model to test the hypothesis that local non-lobar atelectasis can act as a stress concentrator, contributing to inflammation and structural alveolar injury in the surrounding healthy lung tissue during mechanical ventilation.

## Materials and methods

Thirty-five male Wistar rats (250 to 340 g) were taken from nursery of the Instituto de Biofísica Carlos Chagas Filho - Rio de Janeiro/Brazil. All animals received care in compliance with the *Principles of Laboratory Animal Care* formulated by the National Society for Medical Research and the *Guiding Principles in the Care and Use of Animals* approved by the Council of the American Physiological Society, USA. The Ethics Committee on the Use of Animals - Health Sciences Centre from the Federal University of Rio de Janeiro approved the study (IBCCF-188-05/16).

### Animal preparation

Animals were anesthetized intraperitoneally (ip) with 0.4 mg/kg of midazolam and 60 mg/kg of ketamine, which were repeated at half the dose, each 30 minutes along the experiment. A tracheotomy was performed with a snugly fitting cannula (1.5 mm ID). An arterial catheter (18 gauge (Ga) × 8 cm, Arrow International, USA) was inserted into the right carotid artery for continuous arterial pressure (AP) monitoring and for blood sampling. At the end of surgical instrumentation, animals were paralyzed (pancuronium bromide, 0.3 mg/kg, intravenously (iv)) and mechanically ventilated (Inspira ASV, Harvard Apparatus, Holliston, MA, USA) using volume-controlled ventilation (VCV) mode, tidal volume (V_T_) of 6 ml/kg, respiratory rate (RR) of 90 breaths/minute, inspiratory to expiratory time ratio (I:E) of 1:2, positive end-expiratory pressure (PEEP) of 0 cmH_2_O and oxygen inspired fraction (F_I_O_2_) of 1.0, which were the baseline settings. The depth of anesthesia was monitored continually by observing arterial blood pressure and heart rate.

### Induction of atelectasis

Atelectasis was induced by bronchial blocking: after five minutes of stabilization and pre-oxygenation with F_I_O_2_ 1.0, a silicon cylinder blocker (diameter 1.9 Fr, and length 1.5 mm) was attached to a catheter metallic guidewire (L-Cath, Becton Dickinson, USA) and inserted through the tracheostomy until wedged in the terminal bronchial tree, where it was released through displacement of the catheter over the guide wire, softly pulling the silicon piece against the lung.

### Ventilation protocols

After bronchial blocking, animals were randomized to one of two groups according to the following mechanical ventilation settings: 1) V_T_ = 10 ml/kg and PEEP = 3 cmH_2_O (V_T_10/PEEP3); and 2) V_T_ = 20 ml/kg and PEEP = 0 cmH_2_O (V_T_20/zero end-expiratory pressure (ZEEP)). We choose these settings to evaluate the injury modulation trough a clearly injurious modality (V_T_20) and a less injurious V_T_ setting (V_T_10). In addition, in the last group we applied PEEP to maintain similar levels of mean airway pressure in both groups. Respiratory rate was 30 breaths per minute, I:E was 1:2, and F_I_O_2_ = 0.5. Six animals in each group were mechanically ventilated for 180 minutes. Instrumental dead space was increased in the V_T_20/ZEEP group to keep similar arterial partial pressure of carbon dioxide (PaCO_2_) levels between groups.

Three series of experiments were performed, for histological analysis (n = 12), for tissue cytokines analysis (n = 12), and for micro computed tomography (microCT) imaging (n = 2). Also, another six non-ventilated healthy animals were used as controls for cytokines and histological studies.

### Histopathology of lung tissues

At the end of the experiment the animals were heparinized and exsanguinated by cutting large abdominal vessels. The trachea was clamped at end-expiration and the lungs were extracted in block, fixed in formalin, and subsequently embedded in paraffin. For all animals we performed the same procedure: the trachea was clamped at the end of the expiration, and all lungs were securely ligated at the level of main bronchi at this same pressure. Transversal slices from the apex to the base were cut (4-μm thick), carefully making sure to pass through the atelectasis. After repeating the same procedure in the contralateral lung without atelectasis, slices were stained with hematoxylin-eosin.

**Figure 1:**
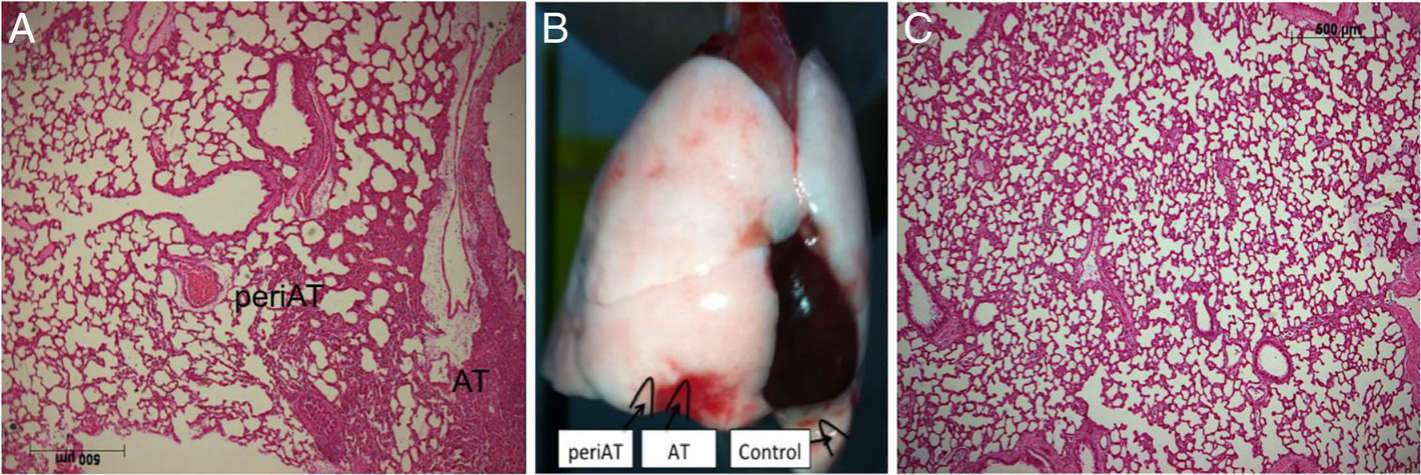
**Histological and** ***ex vivo*** **images. (A)** Histological images (50×) of the atelectasis (AT) and peri-atelectasis (PeriAT) regions are exhibited. Note the alveolar hyperinflation of the surrounding alveoli. **(B)** *Ex vivo* image of the lungs exhibiting the AT, PeriAT, and the control regions of interest. **(C)** Histological image of the contralateral lung (control region) of the same animal is exhibited. Note the difference in surface area of alveoli when compared with the PeriAT region.

Airspace injury was evaluated in a blinded fashion, by a semiquantitative method that measured alveolar disruption, neutrophilic infiltration, edema and hemorrhage as previously described [21,22]. Each one of these variables was scored from 0 to 3 points according to the severity of the changes (0 = none, 1 = mild, 2 = moderate, and 3 = severe). First, we defined the following regions of interest (ROIs): atelectasis (AT) was defined as the alveolar collapse region (confirmed by direct observation of the blocker during the tissue sampling); peri-atelectasis (PeriAT) was defined as the tissue portion 3 mm adjacent to AT, and control was defined as the anterior portion of the inferior lobe of the contralateral lung (Figure 1). Two slices from each lung were analyzed, randomly observing ten fields (400× magnification) of each ROI.

The scoring system was validated by review of selected sections together with a veterinary pathologist.

### Lung morphometry

**Figure 2:**
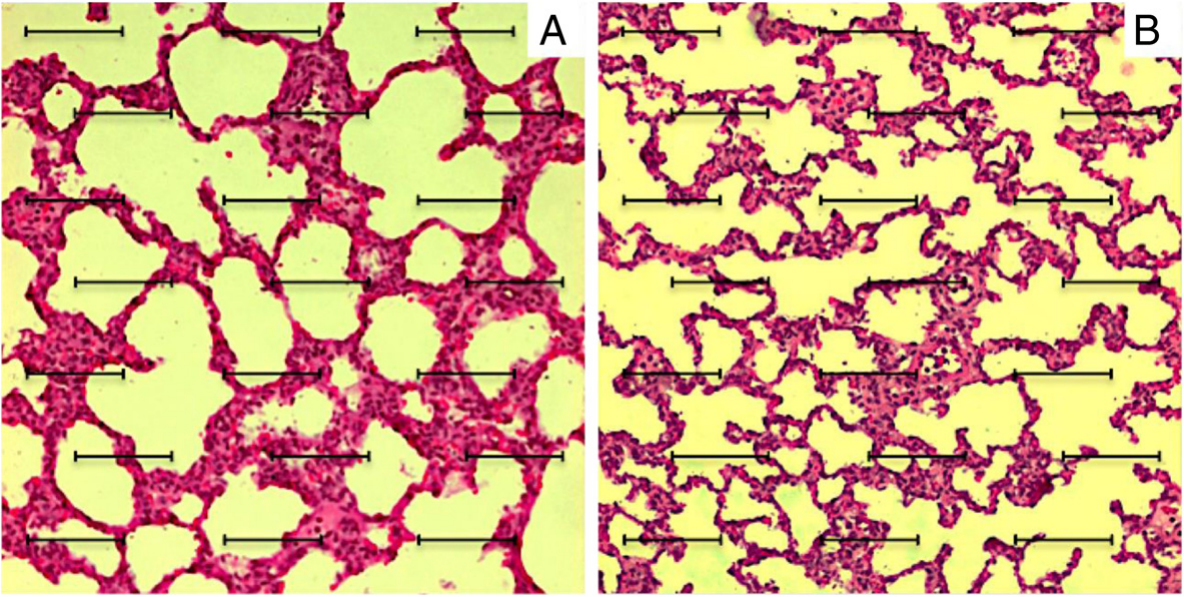
**Volume/surface (V/S) ratio grid.** Representative images (200×) from histological preparation of an animal of tidal volume = 20 ml/kg and positive end-expiratory pressure = 0 cmH_2_O group. The grid used for calculation of the V/S ratio is superimposed on the fields. **A** Corresponds to peri-atelectasis, and **B** corresponds to control lung region.

To determine the inflation condition of alveoli from PeriAT and control region, we used the volume-to-surface (V/S) ratio, a morphometric technique described by Weibel *et al*. [23]. A microscope field was projected in a screen and a grid superimposed on the field. The grid consisted of 21 test lines equivalent to 100-μm in length at the magnification used. The relative volume of the lung occupied by alveoli is equal to the percentage of points falling within alveoli (alveolar hits). The surface area of the alveoli is proportional to the number of times that test lines intersect the alveolar walls, and inversely proportional to the length of the lines (Figure 2). The V/S ratio of the alveoli may then be calculated as follows [24]:

$$ \mathrm{V}/\mathrm{S} = \mathrm{length}\ \mathrm{of}\ \mathrm{test}\ \mathrm{lines} \times \mathrm{times}\ \mathrm{of}\ \mathrm{alveolar}\ \mathrm{hits}\ /\ 4 \times \mathrm{times}\ \mathrm{alveolar}\ \mathrm{intersections} $$

### Protein assays

Tissue samples from the three ROIs (AT, PeriAT, and control lung) were collected at the end of the ventilation protocols. They were identified and cut under direct vision, and immediately frozen and homogenized. Quantification of IL-1β and cytokine-induced neutrophil chemoattractant (CINC-1) was performed using ELISA with high sensitivity kits (R&D Systems Inc, Minneapolis, MN, USA) in accordance with the manufacturer’s instructions.

### Micro-computed tomography (microCT)

To better characterize the atelectasis we acquired two series of microCT images in two animals. One was made after 30 minutes and the other one after three hours of the atelectasis induction. The microCT protocol was performed with 75 kV and 145 μA, 1.5 magnification, and three frames of 1,024 projections. The reconstruction was done with a body filter and gave a volume with a slice separation of 0.48 mm, 1024 × 1024 matrix, and recon voxel size of 0.170 mm.

### Statistical analysis

Variables were tested for normality with the Shapiro-Wilk test. For normally distributed variables, we used repeated-measures analysis of variance (ANOVA) for the comparison of any variable collected multiple times during the protocol. The Bonferroni adjustment for multiple tests was applied for post hoc comparisons. When the assumption of normality was not met, we used the Friedman test as the non-parametric alternative to the repeated measures ANOVA. Pair-wise planned contrasts were performed using the Bonferroni correction for multiple comparisons. To compare non-paired samples we used the Kruskal-Wallis test. All statistical tests were two-tailed, and the significance was set at *P* <0.05.

## Results

Both ventilatory strategies presented hemodynamic stability and all animals survived until the end of the experiment. Baseline gas exchange is show in Table 1. Respiratory system mechanics and hemodynamic data over the protocol are shown in Table 2.

**Table 1:** **Baseline arterial blood gases of the two mechanical ventilation and the control groups**

	**pH**	**pCO**_**2**_	**pO**_**2**_	**HCO**_**3**_	**SatO**_**2**_	**pO**_**2**_ **/FiO**_**2**_
**VT20/ZEEP**	7.41 (0.05)	43.7 (5.7)*	414 (180)*	28.5 (3.8)	99 (0.2)	414 (182)
**VT10/PEEP3**	7.18 (0.03)*	77.6 (15.3)*	215 (60)*	29.3 (4.3)	99 (1.4)	215 (60)*
**Control group**	7.36 (0.04)	54.3 (5)*	99 (8.9)*	30.7 (1.5)	97 (0.6)	473 (42)

VT20/ZEEP: tidal volume = 20 mL/kg and zero end-expiratory pressure (PEEP = 0 cmH_2_O); VT10/PEEP3: tidal volume = 10 mL/kg and positive end-expiratory pressure = 3 cmH_2_O. Data are expressed as mean (standard deviation). *P < 0.05. PaO_2_/F_I_O_2_: ratio of partial pressure of arterial oxygen to the fraction of inspired oxygen.

**Table 2:** **Ventilatory and hemodynamic variables during the three hours of mechanical ventilation**

**VT20/ZEEP**	Vt/kg (mL/L)*	PEEP (cmH2O)*	Ppeak (cmH2O)*	Pmean (cmH2O)*	Rrs*	Ers*	**Syst AP(mmHg)**	**Diast AP (mmHg)**	**Mean AP (mmHg)**
**Baseline**	19.5 (0.5)	0.01 (0.04)	19.5 (1.8)	4.7 (0.3)	0.22 (0.02)	2.77(0.4)	150 (37)	126 (31)	138 (34)
**30 min**	19.4 (0.7)	0.01 (0.02)	19.6 (1.7)	4.7 (0.29)	0.21 (0.03)	2.80 (0.4)	147 (31)	125 (26)	136 (27)
**60 min**	19.2 (0.6)	0.01 (0.02)	19.5 (1.6)	4.7 (0.29)	0.21 (0.02)	2.84 (0.4)	142 (30)	120 (26)	131 (27)
**90 min**	19.2 (0.8)	0.01 (0.02)	19.5 (1.7)	4.7 (0.29)	0.21 (0.03)	2.84 (0.4)	147 (25)	125 (21)	137 (22)
**120 min**	19.1 (0.8)	0.01 (0.01)	19.4 (1.5)	4.3 (0.27)	0.21 (0.03)	2.86 (0.4)	133 (29)	113 (26)	124 (27)
**150 min**	19.4 (1)	0.02 (0.02)	19.4 (1.5)	4.7 (0.23)	0.20 (0.03)	2.82 (0.4)	125 (28)	106 (27)	116 (27)
**180 min**	19.4 (1)	0.02 (0.02)	19.4 (1.5)	4.7 (0.23)	0.20 (0.02)	2.83 (0.4)	125 (27)	106 (26)	116 (26)
**VT10/PEEP3**									
**Baseline**	9.6 (0.3)	3.3 (0.4)	16.5 (1.7)	6.0 (0.4)	0.31 (0.08)	4.03 (0.5)	152 (30)	128 (26)	140 (27)
**30 min**	9.5 (0.9)	3.3 (0.4)	16.6 (1.3)	6.0 (0.4)	0.30 (0.08)	4.13 (0.5)	131 (21)	111 (27)	121 (26)
**60 min**	9.5 (1.2)	3.3 (0.4)	16.8 (1)	6.1 (0.3)	0.32 (0.17)	4.18 (0.5)	130 (12)	109 (14)	120 (13)
**90 min**	9.6 (1.2)	3.3 (0.4)	16.3 (1.5)	6.0 (0.4)	0.28 (0.05)	4.07 (0.7)	124 (19)	104 (20)	114 (20)
**120 min**	9.6 (1.2)	3.3 (0.4)	16.4 (1.3)	6.0 (0.4)	0.28 (0.05)	4.15 (0.6)	127 (20)	106 (19)	117 (19)
**150 min**	9.5 (1.2)	3.3 (0.4)	16.3 (1.2)	6.0 (0.4)	0.26 (0.04)	4.15 (0.6)	116 (16)	96 (17)	107 (16)
**180 min**	9.5 (1.1)	3.3 (0.5)	16.4 (1.0)	6.0 (0.3)	0.27 (0.04)	4.22 (0.5)	117 (24)	95 (25)	106 (25)

VT20/ZEEP: tidal volume = 20 mL/kg and zero end-expiratory pressure (PEEP = 0 cmH_2_O); VT10/PEEP3: tidal volume = 10 mL/kg and positive end-expiratory pressure = 3 cmH_2_O. Rrs = resistance of the respiratory system (cmH_2_O/L/s); Ers = elastance of the respiratory system (cmH_2_O/L). Data are expressed as mean (standard deviation). *P < 0.05.

We choose the ventilator-group settings to evaluate the injury modulation trough a clearly injurious modality (V_T_20) and a less injurious V_T_ setting (V_T_10), but the V_T_10 group resulted in a mean airway pressure 25% higher and in a respiratory system elastance 33% higher than the V_T_20 group. Also, the stress index was >1.2 in the V_T_10 group.

**Figure 3:**
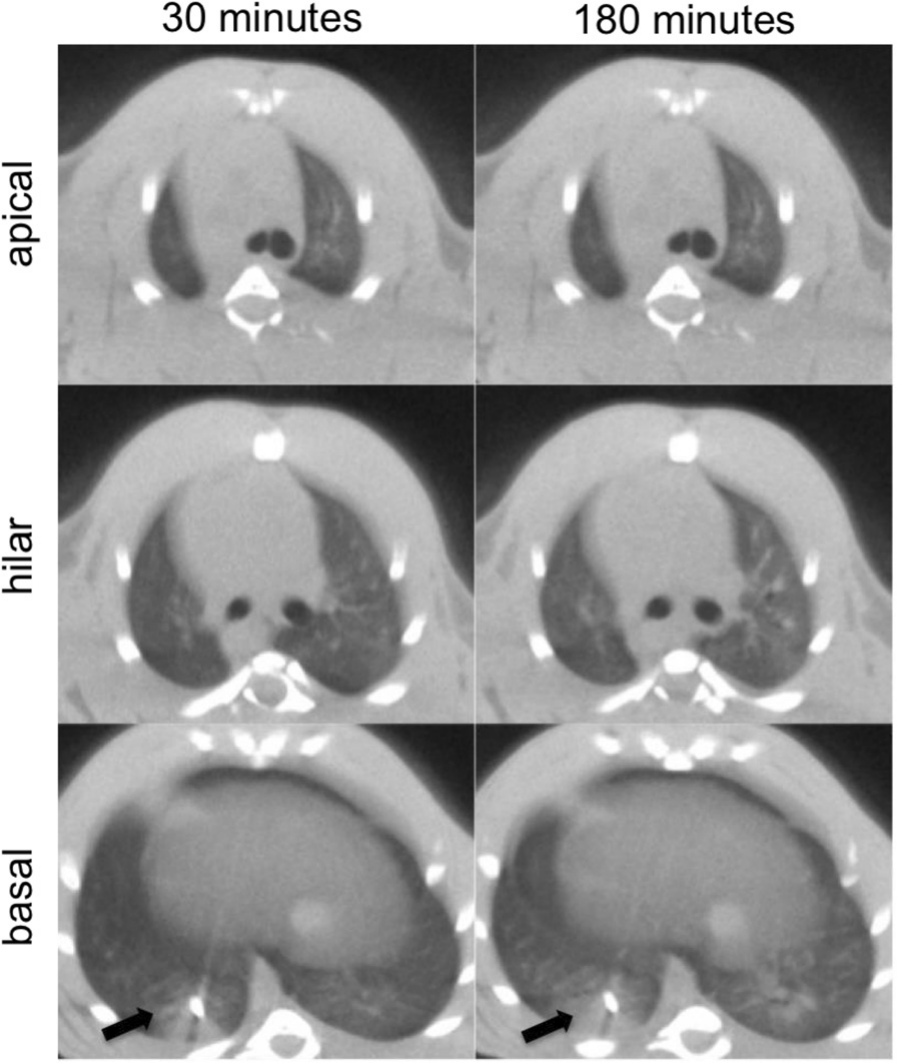
**Micro computed tomography (MicroCT) images.** MicroCT images over time of one representative animal of tidal volume = 20 ml/kg and positive end-expiratory pressure = 0 cmH_2_O group are exhibited. The black arrow indicates the atelectasis and the blocker.

Atelectasis was successfully induced in the basal region of the lung of almost all animals (26/29 animals). The microCT images of the animals revealed that the volume of the atelectasis was 0.12 and 0.21 cm^3^, respectively (Figure 3). The microCT images demonstrated that there was not any other lung collapse at the start of the protocol.

### Histological analysis

**Figure 4:**
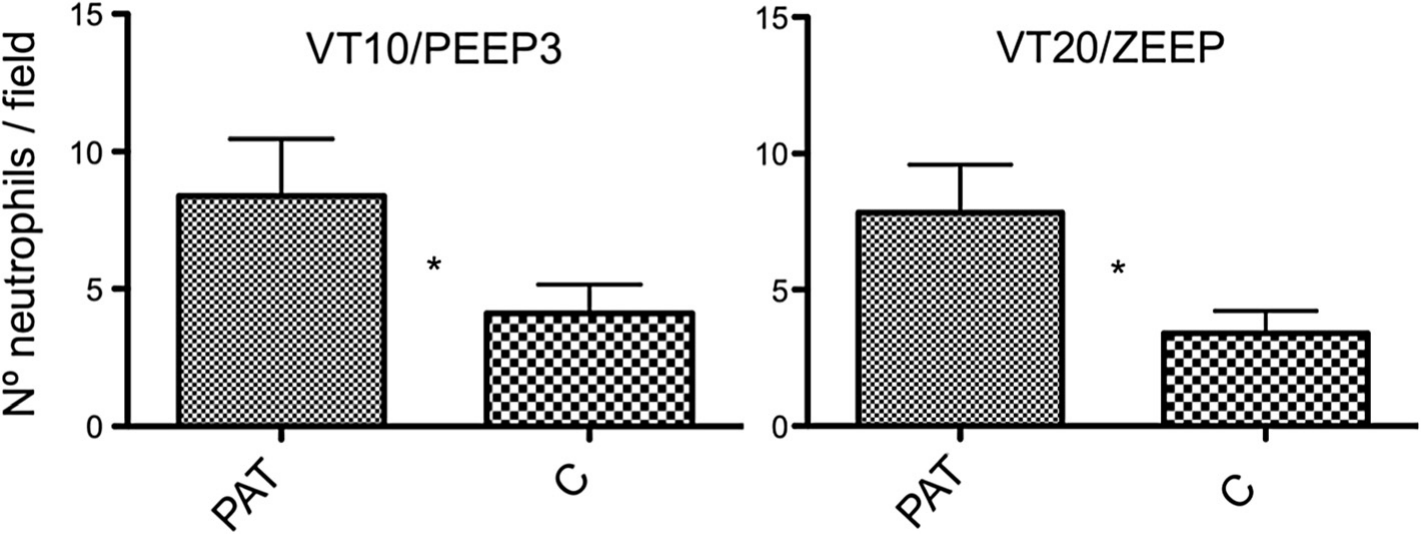
**Neutrophilic infiltration.** Number of neutrophils/field (400×) of the two ventilatory groups are exhibited: Group 1: tidal volume (V_T_) = 10 ml/kg and positive end-expiratory pressure (PEEP) = 3 cmH_2_O (V_T_10/PEEP3). Group 2: V_T_ = 20 ml/kg and PEEP = 0 cmH_2_O (V_T_20/zero end-expiratory pressure (ZEEP)). The results of the peri-atelectasis region (PeriAT) versus the contralateral lung region (C) are exhibited. Data are presented as medians and ranges. *P <0.05.

Atelectasis was evident in the lung samples of all animals analyzed and was consistent with the *ex vivo* macroscopic confirmation. The microscopic analysis of the region inside the atelectasis did not show any alterations suggestive of alveolar disruption; however, it showed a large amount of intravascular neutrophils and vascular congestion. Alveolar disruption and neutrophilic infiltration were higher in the PeriAT region than the corresponding contralateral lung control (Figure 4). Edema was higher in the PeriAT region compared to the control in the V_T_20 group, but did not reach statistical difference in the V_T_10 group (*P* = 0.06). Contrasting the same corresponding ROIs, there were no greater differences between V_T_10/PEEP3 and V_T_20/ZEEP groups (Table 3).

**Table 3:** **Histologic findings**

**VT20/ZEEP**	**Peri-atelectasis**	**Control**	***P*** **-value**
Alveolar disruption	1.3 (0.7 to 2.2)	1 (0.6 to 1.2)	<0.05
Neutrophil infiltration	3 (2 to 3)	1 (1 to 3)	<0.05
Interstitial edema	2.2 (1.4 to 2.6)	1.1 (0.9 to 1.2)	<0.05
Hemorrage	1.5 (0.9 to 2)	1.1 (1 to 2)	0.6
**VT10/PEEP3**			
Alveolar disruption	1.7 (1 to 2.2)	1.1 (0.9 to 1.2)	<0.05
Neutrophil infiltration	3 (2 to 3)	2 (1 to 2)	<0.05
Interstitial edema	2.4 (1.7 to 2.7)	1.1 (0.8 to 2.1)	0.06
Hemorrage	1.2 (1.1 to 2)	1.7 (0.8 to 2)	0.6
**TOTAL**			
Alveolar disruption	1.4 (0.7 to 2.2)	1 (0.6 to 1.2)	<0.05
Neutrophil infiltration	3 (1 to 3)	1.5 (3 to 1)	<0.05
Interstitial edema	2.3 (1.4 to 2.7)	1.1 (0.8 to 2.1)	<0.05
Hemorrage	1.5 (0.9 to 2)	1.4 (0.8 to 2)	0.6

VT20/ZEEP: tidal volume = 20 mL/kg and zero end-expiratory pressure (PEEP = 0 cmH_2_O); VT10/PEEP3: tidal volume = 10 mL/kg and positive end-expiratory pressure = 3 cmH_2_O. Peri-atelectasis and Control represent peri-atelectasis and contralateral lung regions, respectively. Data are expressed as median and range.

**Figure 5:**
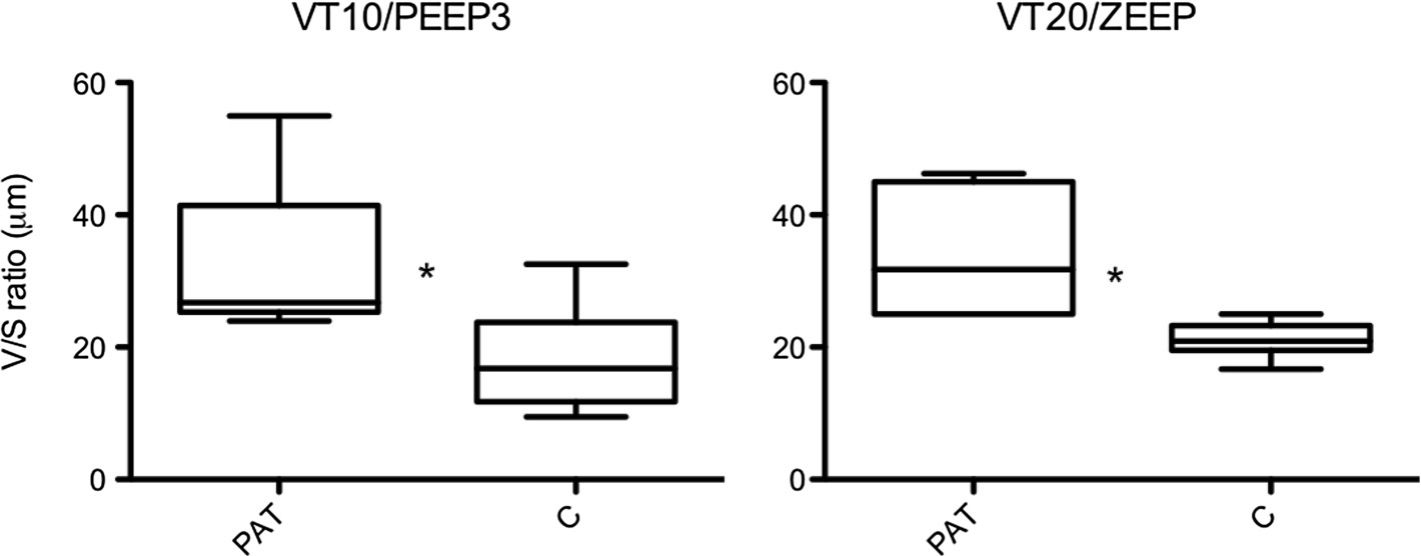
**Volume/surface (V/S) ratio.** V/S of the two ventilatory groups are exhibited: Group 1: Tidal volume (V_T_) = 10 ml/kg and positive end-expiratory pressure (PEEP) = 3 cmH_2_O (V_T_10/PEEP3). Group 2: V_T_ = 20 ml/kg and PEEP = 0 cmH_2_O (V_T_20/zero end-exiratory (ZEEP)). The results of the peri-atelectasis region (PeriAT) versus the contralateral lung region (C) are exhibited. Data are presented as medians and ranges. *P <0.05.

The V/S ratio was higher in the PeriAT region than the corresponding contralateral lung (control), demonstrating that the PeriAT region (*P* <0.05) presented more distension of alveoli. There were no differences in V/S ratio in the PeriAT region between V_T_10/PEEP3 and V_T_20/ZEEP groups (Figure 5).

**Figure 6:**
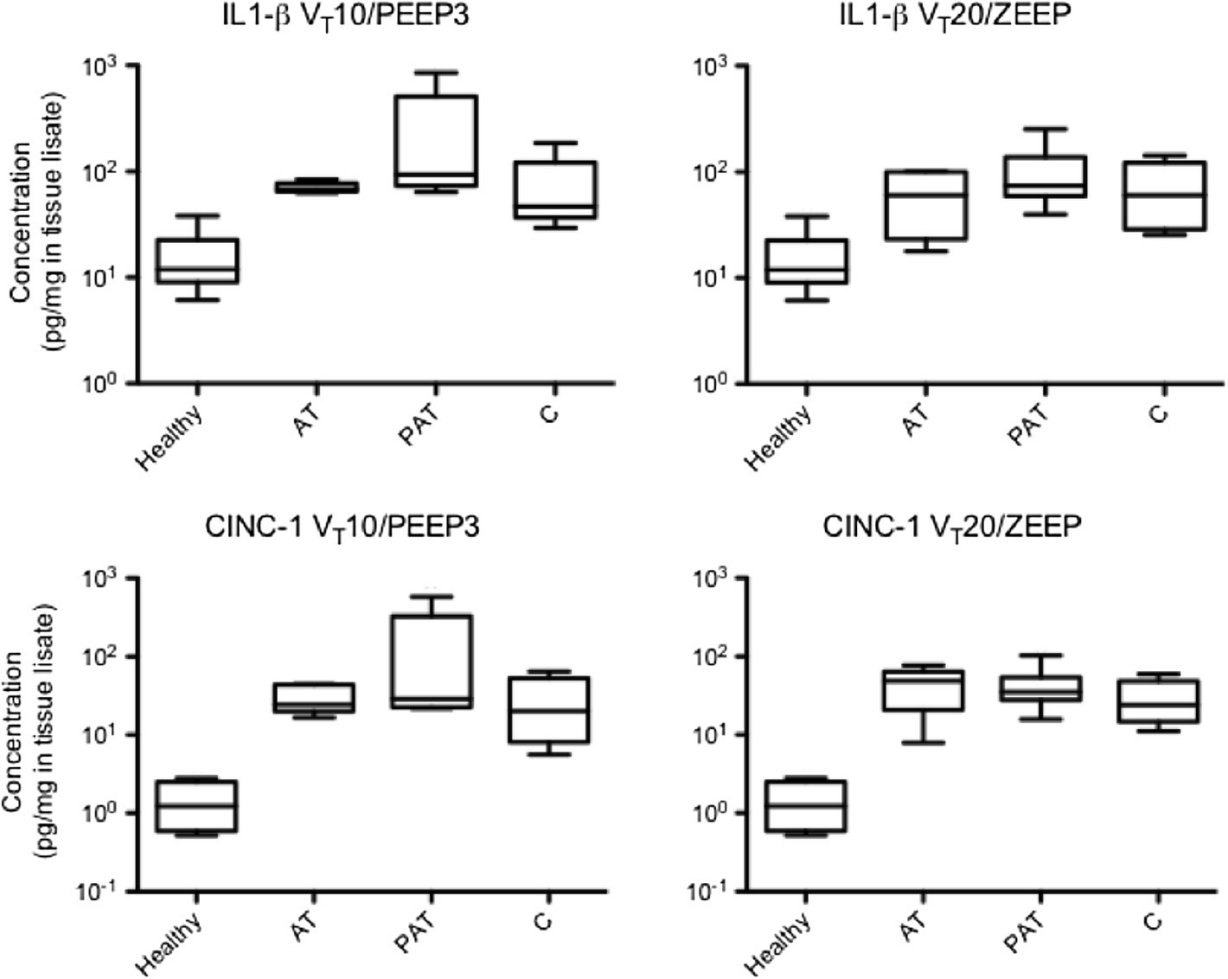
**Expression of cytokine-induced neutrophil chemoattractant (CINC-1) and IL-1β proteins.** Expression of CINC-1 and IL-1β proteins of the two ventilatory groups are exhibited: Group 1: Tidal volume (V_T_) = 10 ml/kg and positive end-expiratory pressure (PEEP) = 3 cmH_2_O (V_T_10/PEEP3). Group 2: V_T_ = 20 ml/kg and PEEP = 0 cmH_2_O (V_T_20/zero end-exiratory (ZEEP)). AT, PeriAT and C represent atelectatic, peri-atelectasis and contralateral lung regions, respectively. Healthy corresponds to the non-ventilated control animals. Data are expressed as medians and ranges.

### Tissue cytokines

We did not find statistical differences between ROIs in tissue IL-1β or cytokine-induced neutrophil chemoattractant (CINC-1) (Figure 6).

## Discussion

In the present study we used a novel model of non-lobar atelectasis through non-selective bronchial blocking in rats, and investigated its effects on the atelectatic area as well as on the surrounding healthy lung tissue during injurious mechanical ventilation. The atelectasis model was reliable and greater histological evidence of hyperinflation and inflammation was observed in the PeriAT region.

Most of the experimental studies on the relationships between atelectasis, mechanical ventilation and VILI have the starting point of surfactant depletion and/or some type of acute lung-insult [25]. For instance, surfactant depletion is usually produced by saline lavage and results in decrease in lung compliance and hypoxemia [26]. When saline lavage is followed by mechanical ventilation with high volumes and low PEEP, a type of lung injury results that is very similar to ARDS in humans [25]. These models invariably contain, from the very beginning, unstable airspaces and a consequent gravitational gradient of collapse. In contrast, our experimental model was designated in such a way that the starting point was, as much as possible, only the primary interaction between collapsed and aerated regions, that is, before any surfactant dysfunction and/or another potential VILI mechanism could become involved. Accordingly, our model initially presented lung parenchyma homogeneously aerated with an isolated peripheral inhomogeneity. In this way we can focus on the true interaction between collapse and healthy alveoli during the mechanical ventilation.

VILI has been thought to predominate in the collapse-dependent regions and/or in the over-distended non-dependent regions. Tsuchida *et al*. demonstrated that in a model of alveolar collapse and surfactant depletion the atelectasis regions *per se* were protected from alveolar damage, whereas alveoli from the aerated regions were more affected [15]. Very recently, Cereda *et al*. [27] used magnetic resonance imaging to provide *in vivo* imaging evidence of airspace expansion in a surfactant-depleted model. Their data suggest that atelectasis could contribute to VILI by reciprocal increases in airspace size and that lung injury could be minimized through alveolar recruitment and adequate PEEP. This suggestion is supported by morphometric [15] and clinical data [28] and contrasts with previous reports suggesting an increase of VILI in the atelectasis during MV [11,12]. Adding another point of view for the pressing question on the localization of the regional onset of lung inflammation, other recent findings suggest that the interface between the collapsed and aerated tissue may play an important role in lung injury [5].

Our findings, together with those of other investigators [29], suggest that the increased susceptibility to VILI is related to small-length-scale heterogeneities of the lung parenchyma. Indeed, Rausch *et al*. [30] estimated, employing synchrotron-based x-ray tomographic microscopy on isolated rat lungs, that local strains developing in alveolar walls are as much as four times higher than the global ones. Their data suggest that thin regions may become overstretched, whereas regions with tissue accumulation remain unchallenged. These data fit with our own results and strongly suggest that a tidal stretch of the healthy aerated parts can play a primary role in the activation of the inflammatory signaling cascade [18,28].

It seems likely that the collapsed region may indirectly damage the surrounding initially-healthy aerated tissue, acting as a stress riser. We found a concentration of mechanical trauma (alveolar wall disruptions and signs of over-distension) and inflammation (neutrophilic infiltration and interstitial edema) in the region surrounding the atelectasis. In their theoretical analysis, Mead *et al*. estimated that the alveolar pressure at the junction of the fully collapsed and expanded alveoli could be as high as 4 to 5 times the applied pressure [6]. This landmark estimation of Mead of approximately four times local amplification was recently confirmed using synchrotron-based x-ray tomographic microscopy in a preparation of rat lungs [30], discussed above. In the same direction, Rouby *et al*. described that in an autopsy study [31], expanded pseudocysts were concentrated around atelectatic lung regions. We found evidence of inflammation in the atelectasis region (intravascular neutrophils and tissue cytokines), however, we did not identify signs of alveolar disruptions in this area. We speculate that it could be due to vascular injury secondary to high blood flow in a systemic inflammatory scenario that could induce endothelial activation and neutrophil adhesion, explaining the inflammatory signs in this non-ventilated portion of the lung.

We did not find significant differences in cytokine concentrations between PeriAT and control regions in the two ventilatory groups. We can postulate two possible explanations for that: 1) the duration of the protocol was too short, and 2) the technique of tissue sampling may have been unable to accurately represent the two ROIs.

The current work has limitations. First, the region of atelectasis was induced exclusively in the basal region of the lung. However, the anterior or posterior location could not be selected by the methodology and setup used. Bronchoscopy-guided plugs applied in larger animals may be a suitable alternative, allowing more topographical options for the induction of atelectasis and better reproduction of clinically relevant atelectasis in healthy lungs. Second, the duration of the ventilatory protocol was 180 minutes. Maybe a longer protocol would allow studying more precisely the inflammatory response patterns and the relationships between the ROIs. Third, a non-lobar atelectasis model was used that is different from the dependent collapse characteristically observed in ARDS patients. However, it served well to assess the spatial and time effects of localized inhomogeneity, without the concomitant presence since the starting point, of other sources of airspaces instabilities. Fourth, although blockers are biocompatible and used in the clinical setting, we cannot ignore the fact that the blockers could induce some degree of airway and or parenchymal trauma. Finally, in an *ex vivo* lung injury model the presence of atelectasis was associated with higher transcription of stress kinases and histological evidence of lung damage in the context of protective mechanical ventilation (low tidal volume) [19]. This finding suggests that a control for future work can be a condition characterized by the presence of atelectasis without mechanical stress (for instance, using a continuous positive airway pressure mode).

## Conclusions

In conclusion, the present findings suggest that local non-lobar atelectasis acts as a stress concentrator, generating structural alveolar injury and inflammation in the surrounding lung tissue.

## Key messages

- Local non-lobar atelectasis acts as a stress concentrator
- Local non-lobar atelectasis generates structural alveolar injury in the surrounding healthy tissue
- Local non-lobar atelectasis generates inflammation in the surrounding healthy tissue

## Abbreviations

ARDS: acute respiratory distress syndrome
AT: atelectasis
CINC-1: cytokine-induced neutrophil chemoattractant
CT: computed tomography
ELISA: enzyme-linked immunosorbent assay
F_I_O_2_: oxygen inspiratory fraction
Fr: French
Ga: gauge
I:E: inspiratory-to-expiratory time ratio
IL-1β: interleukine-1β
PEEP: positive end-expiratory pressure
PeriAT: peri-atelectasis
ROI: region of interest
RR: rRespiratory rate
V/S ratio: volume-to-surface ratio
VCV: volume-controlled ventilation
VILI: ventilator-induced lung injury
V_T_: tidal volume
ZEEP: zero end-expiratory pressure

## References

[1] Grasso S, Stripoli T, Sacchi M, Trerotoli P, Staffieri F, Franchini D, De Monte V, Valentini V, Pugliese P, Crovace A, Driessen B, Fiore T (2009). Inhomogeneity of lung parenchyma during the open lung strategy: a computed tomography scan study. Am J Respir Crit Care Med.

[2] Gattinoni L, Caironi P, Cressoni M, Chiumello D, Ranieri VM, Quintel M, Russo S, Patroniti N, Cornejo R, Bugedo G (2006). Lung recruitment in patients with the acute respiratory distress syndrome. N Engl J Med.

[3] Pinhu L, Whitehead T, Evans T, Griffiths M (2003). Ventilator-associated lung injury. Lancet.

[4] Gattinoni L, Carlesso E, Langer T (2012). Towards ultraprotective mechanical ventilation. Curr Opin Anaesthesiol.

[5] Cressoni M, Cadringher P, Chiurazzi C, Amini M, Gallazzi E, Marino A, Brioni M, Carlesso E, Chiumello D, Quintel M, Bugedo G, Gattinoni L (2014). Lung inhomogeneity in patients with acute respiratory distress syndrome. Am J Respir Crit Care Med.

[6] Mead J, Takishima T, Leith D (1970). Stress distribution in lungs: a model of pulmonary elasticity. J Appl Physiol.

[7] Baumgardner JE (2011). New images, new insights for VILI. J Appl Physiol.

[8] Yoshida T, Torsani V, Gomes S, De Santis RR, Beraldo MA, Costa ELV, Tucci MR, Zin WA, Kavanagh BP, Amato MBP (2013). Spontaneous Effort Causes Occult Pendelluft during Mechanical Ventilation. Am J Respir Crit Care Med.

[9] Slutsky AS, Ranieri VM (2013). Ventilator-induced lung injury. N Engl J Med.

[10] Dreyfuss D, Saumon G (1998). Ventilator-induced lung injury: lessons from experimental studies. Am J Respir Crit Care Med.

[11] Otto CM, Markstaller K, Kajikawa O, Karmrodt J, Syring RS, Pfeiffer B, Good VP, Frevert CW, Baumgardner JE (2008). Spatial and temporal heterogeneity of ventilator-associated lung injury after surfactant depletion. J Appl Physiol.

[12] de Prost N, Costa EL, Wellman T, Musch G, Winkler T, Tucci MR, Harris RS, Venegas JG, Vidal Melo MF (2011). Effects of surfactant depletion on regional pulmonary metabolic activity during mechanical ventilation. J Appl Physiol.

[13] Muscedere JG, Mullen JB, Gan K, Slutsky AS (1994). Tidal ventilation at low airway pressures can augment lung injury. Am J Respir Crit Care Med.

[14] Tremblay L, Valenza F, Ribeiro SP, Li J, Slutsky AS (1997). Injurious ventilatory strategies increase cytokines and c-fos m-RNA expression in an isolated rat lung model. J Clin Invest.

[15] Tsuchida S, Engelberts D, Peltekova V, Hopkins N, Frndova H, Babyn P, McKerlie C, Post M, McLoughlin P, Kavanagh BP (2006). Atelectasis causes alveolar injury in nonatelectatic lung regions. Am J Respir Crit Care Med.

[16] ARDSNet (2000). Ventilation with lower tidal volumes as compared with traditional tidal volumes for acute lung injury and the acute respiratory distress syndrome. N Engl J Med.

[17] Terragni PP, Rosboch G, Tealdi A, Corno E, Menaldo E, Davini O, Gandini G, Herrmann P, Mascia L, Quintel M, Slutsky AS, Gattinoni L, Ranieri VM (2007). Tidal hyperinflation during low tidal volume ventilation in acute respiratory distress syndrome. Am J Respir Crit Care Med.

[18] Borges JB, Costa ELV, Suarez-Sipmann F, Widström C, Larsson A, Amato M, Hedenstierna G (2014). Early inflammation mainly affects normally and poorly aerated lung in experimental ventilator-induced lung injury*. Crit Care Med.

[19] Fanelli V, Mascia L, Puntorieri V, Assenzio B, Elia V, Fornaro G, Martin EL, Bosco M, Delsedime L, Fiore T, Grasso S, Ranieri VM (2009). Pulmonary atelectasis during low stretch ventilation: “open lung” versus “lung rest” strategy. Crit Care Med.

[20] Albert RK (2012). The role of ventilation-induced surfactant dysfunction and atelectasis in causing acute respiratory distress syndrome. Am J Respir Crit Care Med.

[21] Hong S-B, Koh Y, Lee I-C, Kim MJ, Kim WS, Kim D-S, Kim WD, Lim C-M (2005). Induced hypothermia as a new approach to lung rest for the acutely injured lung. Crit Care Med.

[22] Kira S, Daa T, Kashima K, Mori M, Noguchi T, Yokoyama S (2005). Mild hypothermia reduces expression of intercellular adhesion molecule-1 (ICAM-1) and the accumulation of neutrophils after acid-induced lung injury in the rat. Acta Anaesthesiol Scand.

[23] Weibel ER, Kistler GS, Scherle WF (1966). Practical stereological methods for morphometric cytology. J Cell Biol.

[24] Glazier JB, Hughes JM, Maloney JE, West JB (1967). Vertical gradient of alveolar size in lungs of dogs frozen intact. J Appl Physiol.

[25] Matute-Bello G, Frevert CW, Martin TR (2008). Animal models of acute lung injury. Am J Physiol Lung Cell Mol Physiol.

[26] Rotta AT, Gunnarsson B, Fuhrman BP, Hernan LJ, Steinhorn DM (2001). Comparison of lung protective ventilation strategies in a rabbit model of acute lung injury. Crit Care Med.

[27] Cereda M, Emami K, Xin Y, Kadlecek S, Kuzma NN, Mongkolwisetwara P, Profka H, Pickup S, Ishii M, Kavanagh BP, Deutschman CS, Rizi RR (2013). Imaging the interaction of atelectasis and overdistension in surfactant-depleted lungs. Crit Care Med.

[28] Bellani G, Guerra L, Musch G, Zanella A, Patroniti N, Mauri T, Messa C, Pesenti A (2011). Lung regional metabolic activity and gas volume changes induced by tidal ventilation in patients with acute lung injury. Am J Respir Crit Care Med.

[29] Wellman TJ, Winkler T, Costa ELV, Musch G, Harris RS, Venegas JG, Vidal Melo MF (2012). Effect of regional lung inflation on ventilation heterogeneity at different length scales during mechanical ventilation of normal sheep lungs. J Appl Physiol.

[30] Rausch SMK, Haberthur D, Stampanoni M, Schittny JC, Wall WA (2011). Local Strain Distribution in Real Three-Dimensional Alveolar Geometries. Ann Biomed Eng.

[31] Rouby JJ, Lherm T, Martin De Lassale E, Poete P, Bodin L, Finet JF, Callard P, Viars P (1993). Histologic aspects of pulmonary barotrauma in critically ill patients with acute respiratory failure. Intensive Care Med.

